# Impact of Sleep Respiratory Disorders on Endothelial Function in Children

**DOI:** 10.1155/2013/719456

**Published:** 2013-12-26

**Authors:** Luigia Brunetti, Ruggiero Francavilla, Pietro Scicchitano, Valentina Tranchino, Maria Loscialpo, Michele Gesualdo, Annapaola Zito, Fara Fornarelli, Marco Sassara, Paola Giordano, Vito Leonardo Miniello, Marco Matteo Ciccone

**Affiliations:** ^1^Department of Biomedicine of the Developmental Age, Pediatric Unit “S. Maggiore”, University of Bari, Piazza G. Cesare 11, 70124 Bari, Italy; ^2^Cardiovascular Diseases Section, Department of Emergency and Organ Transplantation (DETO), University of Bari, Piazza G. Cesare 11, 70124 Bari, Italy

## Abstract

Obstructive sleep apnea syndrome (OSAS) in children can induce endothelial dysfunction, a well-known early marker of atherosclerosis. The study aimed to evaluate a link among endothelial function (measured by flow-mediated vasodilation (FMD)), obesity (evaluated by body mass index (BMI)), and sleep disordered breathing (SDB), assessed with apnoea/hypopnoea index (AHI), in a paediatric population. We demonstrated that our little OSAS patients showed an impaired endothelial function as compared to controls. In particular, the higher the AHI, the worst the FMD values and thus the endothelial function. Although the population sample is small, this study demonstrated that OSAS could impair endothelial function and worsen cardiovascular risk profile since childhood.

## 1. Introduction

Obstructive sleep apnoea syndrome (OSAS) is defined as “an intrinsic sleep disturbance characterized by the appearance of repeated episodes of upper airways obstruction (apnoeas) occurring during sleep, usually associated with a reduction in oxygen blood saturation” [1]. The clinical symptoms and polysomnographic characteristics of OSAS in childhood are remarkably different from those in adults [2, 3].

Sleep-disordered breathing (SDB) exists in a continuous spectrum from snoring to severe OSAS. Primary snoring, a common finding in childhood, is not associated with apnoea, oxygen desaturation, or hypoventilation, although snoring may precede obstructive SDB many years before. Epidemiologic childhood data about SDB, and OSAS in particular, are limited [4, 5]. Their incidence is 1–3% from 6-month- to 6-year-old children [4], whilst prevalence is 2–4 fold higher in black population and in premature children [5].

We recently reported the prevalence of this condition in a large Italian children cohort [6]. Nocturnal polysomnography is useful to grade disease severity [7]. Literature studies [8, 9] demonstrated that untreated OSAS leads to an increase in metabolic and cardiovascular problems. Furthermore, SDB incidence in pathologically obese patients is 12–30 times higher than general population, and body mass index (BMI) is the most important risk factor for OSAS [10].

We know that OSAS leads to various cardiovascular and metabolic dysfunctions (inflammatory cytokines up-regulation [8]) maybe caused by intermittent chronic hypoxia and oxidative stress which induces reactive oxygen radicals creation, inflammatory cytokines release, and endothelial dysfunction [8] and, at least, leads to the dangerous “metabolic syndrome” [9, 11]. Even in obese children OSAS could impair their insulin resistance leading to glycaemia levels deregulations, a known condition able to increase cardiovascular risk profile [12, 13], although literature studies are not able to share the weight of OSAS and that of BMI on determination of such a deregulation.

The aim of the study was to evaluate the link among endothelial function, measured by flow-mediated vasodilation (FMD), SDB (evaluated with apnoea/hypopnoea index (AHI)), and BMI in children suffering with SDB.

## 2. Matherials and Methods

This study had been managed in agreement with the standard protocol adopted in our clinic in a previous larger epidemiologic survey about SDB in childhood in Southern Italy managed in 2001 [6].

We considered the paediatric student population of our territory from February 2011 to February 2012 and we admitted them to the Outpatient Pulmonology Clinic Paediatric Unit, Policlinico di Bari, Bari, Italy, in order to screen them for OSAS. The study followed the same criteria already established in our previous work [6].

This cross-sectional prevalence study was designed in two distinct phases: a screening phase, which aimed to select children with a history suggestive of SDB, and a second investigative phase.

All procedures used were in accordance with the guidelines of the Helsinki Declaration on Human Experimentation. The study was carried out in accordance with the Provincial Education Office of Bari and the local school Ethics Committee. Informed consent at enrolment was obtained from all parents and assent was obtained even from the child after full explanation of the procedures.

### 2.1. Screening Phase

A 41-item multiple-choice questionnaire was distributed to children at school. It was formulated according to Brouillette's guidelines revised by Carroll et al. [14–16].

The questionnaire inquired about family history of atopy, child clinical history, snoring and/or apnoeas (frequency and duration) presence, symptoms that could be related to SDB (troubled sleep, enuresis, thirst, sweating, oral breathing, need of afternoon rest, daily sleepiness, poor school achievement), and smoking habits in the family and during pregnancy (>20 cigarettes/day). In the sleep section, questions were structured to score the severity of sleep-related symptoms according to a four-point scale [6].

All the items were statistically and finally resembled into the following:

- scores = 3 (always): habitual snorers (HS);
- scores = 2 (sometimes): occasional snorers (OS);
- scores = 1 (seldom) or 0 (never): non-snorers (NS).

Families that did not return the questionnaires were contacted by phone on two separate occasions (15 days apart) before being classified as nonresponders.

### 2.2. Second Phase

#### 2.2.1. Population

A randomized cohort of children suffering of SDB and a control group entered the second phase of the study. 23 children, mean age 9 ± 3 years, suffering from SDB (group 1), and 32 controls (group 2, mean age 10 ± 3 years) were recruited. Patients younger than 5 years old were excluded because they were supposed to be noncooperative in managing FMD examination. This reduced number of patients included in the study is due to the relative small number of young patients suffering from OSAS in our zone, as already outlined in previous literature data [6, 17]. Furthermore, we included a control group endowed with similar characteristics (age, gender, and BMI) of the patients in order to reduce biases. This means that the two groups did not differ for age, gender, and BMI: this condition is fundamental in order to eliminate confounding factors in our evaluation of FMD measurements. The control group was considered as neither suffering from OSAS nor snoring, with age >5 years old and should be collaborative enough in order to undergo FMD evaluation.

This control group did not undergo polysomnography examination due to its lower questionnaire values (score equals 0 or 1) because such a score reduces the possibility of them to be suffering from OSAS [18].

All children were not under continuous positive air pressure (CPAP) or pharmacological treatment for their disease at enrolment.

Exclusion criteria were: cardiovascular diseases, liver diseases, gastrointestinal diseases, kidneys diseases, severe respiratory insufficiency, and nervous/malformative conditions related to SDB.

All children underwent accurate anamnesis and clinical examination.

#### 2.2.2. Polysomnography

Children suffering from SDB underwent an instrumental definition of the disorder for the diagnosis of either OSAS or primary snoring. Instrumental tests were undertaken while the children had no upper-airway infections.

A device (Vitalog HMS 5000, Pocket Polygraph; Markos srl, Monza, Italy) was used for a nighttime home evaluation. According to the American Sleep Disorders Association's screening methodologies [19], the following parameters were monitored: (1) transcutaneous saturation of oxygen, (2) heart rate (through ECG), (3) snoring, and (4) body position. A microphone was positioned over the larynx for snoring evaluation.

Following the Italian guidelines for the diagnosis of childhood OSAS, recordings with an oxygen desaturation index (ODI) (ODI = the number of significant desaturations [Δ > 4%] per hours of sleep) [20] were adopted to achieve OSAS diagnosis. ODI values were obtained during nocturnal polysomnographic monitoring (NPM) [21, 22]. The NPM was recorded in the sleep laboratory of our unit.

Polysomnography was performed overnight by Vitalog HMS 5000. The conditions of measurements and the parameters monitored are described elsewhere [6].

Obstructive apnoea was defined as absent airflow in the presence of respiratory effort for at least two respiratory cycle times, accompanied by at least a 4% decrease in arterial oxygen saturation.

Obstructive hypopnoea was defined as a decrease of at least 50% in the amplitude of the oronasal thermistor signal, with maintained respiratory effort for at least two respiratory cycle times, accompanied by a >4% decrease in arterial oxygen saturation [21, 22]. The AHI was calculated as number of obstructive apnoea events plus number of obstructive hypopnoea events per hour of sleep time. Children with an AHI > 1 received a diagnosis of mild-to-severe OSAS [20]. In particular, in agreement with literature studies [23, 24], we were more aggressive in OSAS classification in children. In fact, beyond adults classification, we further divided the mild OSAS degrees into a minimum one and a full mild one. In fact, we defined [20]: (a) snoring when AHI was <1; (b) minimum OSAS when AHI value was ≥1 and <3; (c) mild OSAS if AHI value was ≥3 and <5; (d) moderate OSAS when AHI value was ≥5 and <10; (e) severe OSAS if AHI value was ≥10. The nadir of oxygen and the percentage of total sleep time with arterial oxygen saturation <90% were also calculated [6].

#### 2.2.3. FMD of Brachial Artery

All patients of Groups 1 and 2 underwent vascular reactivity measurement of macrocirculation. The study was performed with the subjects fasting for at least 8–12 hours, in a quiet air-conditioned room (22–24°C). Moreover, the subjects were asked not to play or exercise or take exciting substances like caffeine, fatty foods, or vitamins, for at least 4–6 hours before the exam. A preliminary scan to explore the anatomy and landmarks was performed, paying particular attention to poor-quality images, the presence of atherosclerotic plaques, calcifications, arterial tortuosity, or kinking. The scan was done at the right humeral artery in a longitudinal section between 5 and 10 cm above the elbow using a 7.0 MHz or higher linear probe. The study was performed using a high-resolution ultrasonograph (Philips Sonos 5500) connected to an image analysis system certified by the Centro Nazionale Ricerche of Pisa (MVE II), setting positivity to the test value at less than 5%. To reduce observer bias, the same physician performed all the ultrasound examinations. With the subject in supine position for at least 10 minutes, the arm was positioned comfortably in order to get good images of the humeral artery, above the antecubital fossa in the longitudinal plane, identifying the part where the anterior and posterior intimal interfaces between the lumen and vessel wall were clear. A sphygmomanometer cuff was placed at the forearm. After 1 minute of flow image baseline acquisition, the artery was occluded by inflating the cuff to a pressure of 50 mmHg above systolic blood pressure (180–200 mmHg) for exactly 5 min. When the cuff is deflated, it induces a short state of high flow (reactive hyperemia in the forearm microcirculation) through the brachial artery to adjust to the reduced downstream resistance caused by the ischemia-induced dilatation. The resulting increased shear stress provides the stimulus for dilatation of the humeral artery. Within 15 seconds from the end of ischemia, the flow rate and the degree of hyperemia were measured. The image of the artery was then recorded continuously for 2-3 minutes after ischemia. Reactive hyperaemia was calculated as the ratio of maximal dilatation after deflation divided by the diameter at baseline, which corresponds to the maximum FMD recovery value. FMD was analyzed as the percentage increase in brachial artery diameter after the application of a pressure stimulus.

## 3. Statistical Analysis

The data are given as mean values ± standard deviation (SD) and categorical variables as frequencies and percentage. Between-group comparisons were made using nonparametric Mann-Whitney *U* test. Frequencies were compared using Fisher's exact test. A *P* value <0.05 was considered statistically significant. The Pearson correlation coefficient was used to estimate the correlations between variables. The statistical analyses were made by using Statistica 6.1 software (StatSoft Inc., Tulsa, OK, USA).

## 4. Results

According to AHI score, the 23 children of SDB group were subdivided into (see also Table 1):

- snoring: 15 (65.2%);
- minimum OSAS: 4 (17.4%);
- mild OSAS: 4 (17.4%).

No patients were suffering from moderate-to-severe OSAS. Our population had been finally composed by patients who not completely fulfilled adult criteria for OSAS. Our paediatric population had been differentiated as above indicated for SDB diseases. Control group was not suffering from snoring or OSAS; thus, no polysomnography data could be outlined for them.

All included patients showed a total sleep time lasting more than 6 hours/night. Nobody showed a total sleep time with arterial oxygen saturation <90%, while the nadir of oxygen was higher than 91%. Furthermore, the number of those were wakeful among our children was less than 3.

SDB and control statistically differ from each other for BMI percentile values (*P* = 0.048) and FMD ones (*P* < 0.001).

**Figure 1:**
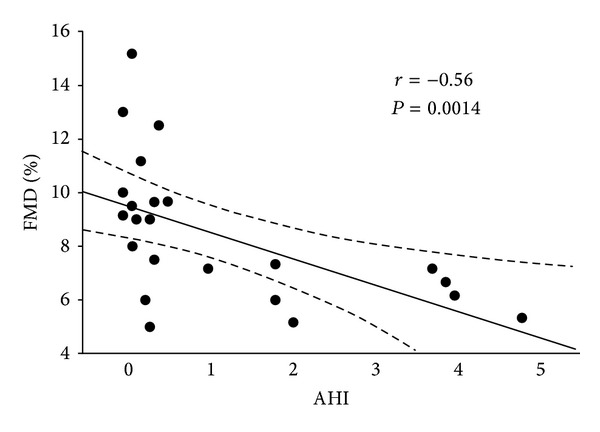
Correlation between FMD% and AHI in Group 1 (*r* = −0.56, *P* = 0.0014).

**Figure 2:**
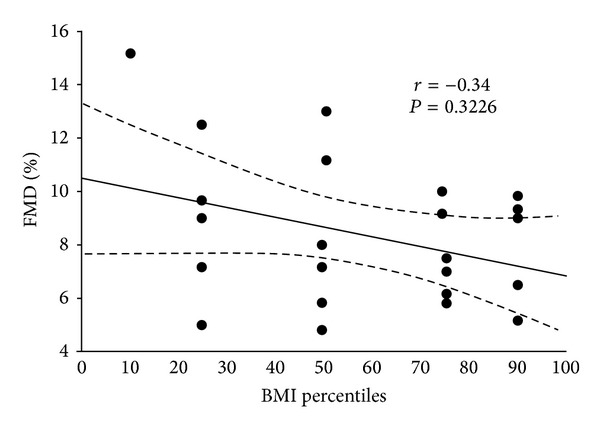
Correlation between FMD% and BMI percentile. (*r* = −0.34, *P* = 0.3226).

Figures 1 and 2 put on evidence of Pearson linear correlation calculated between FMD values and AHI ones, and between FMD and BMI percentiles. It is possible to observe a negative correlation among the same variables: *r* = −0.56, *P* = 0.0014 (comparison between FMD and AHI) and *r* = −0.34, *P* = 0.3226 (comparison between FMD and BMI percentiles), although the latter is not statistically significant.

This means that there is a progressive reduction in FMD values with the worsening of AHI ones. For this reason, the deterioration of children respiratory picture related to their basic pathology induces a parallel worsening of endothelial function evaluated using FMD technique.

## 5. Discussion

About 9% of women and 24% of men in adult population suffer from SDB. Adult percentage of OSAS patients, the most severe being SDB form, is 2–4%, while near to 2-3% pointed out in children [25].

Considering SDB complications such as metabolic alterations (i.e., obesity/diabetes mellitus), dyslipidemia, arterial hypertension, and ischemic cardiomyopathy [26, 27], an early diagnosis of SDB should be performed since early period of life.

It would be important to identify predictors of OSAS. Previous studies outlined that an OSAS has been more commonly associated with snoring [28], troubled sleep, nocturnal sweating, oral breathing, poor school achievements, and daily sleepiness [6], while the role of enuresis is still controversial [29].

Although the first cause of adulthood OSAS is obesity, this illness seems to be due to other causes in paediatric population, such as adenoidal-tonsillar hypertrophy. Nevertheless, even the increased obese children number induced an augmentation of paediatric OSAS incidence and an amplification of its manifestations and cardiovascular complications [30].

The association of obesity with sleep-associated respiratory disturbances, which has traditionally been described as a problem in adults, actually originates in childhood. Brunetti et al. documented in a population of 809 children that frequency of habitual snore was significantly higher in obese children than in overweight and normal-weight subjects (12.5% versus 5.8% versus 4.6%, resp.; *P* = 0.02) [23]. Furthermore, a phenotypic variant of OSAS in children, similar to adults' form, emerged [31].

OSAS could directly worsen endothelial function since early childhood, as recent studies had put on evidence [32], maybe due to an impairment between sympathetic and parasympathetic branches [24]. OSAS increases endothelin-1 plasma levels, which induce could increase arterial stiffness and blood pressure [33].

Even oxygen radicals and all the inflammatory mechanisms linked to OSAS could lead to endothelial dysfunction cause of their negative role on arterial wall remodelling [8], independently from the obesity children conditions and rather associated to the obstruction of upper airways in particular [34, 35].

Kheirandish-Gozal et al. [36] put on evidence of poor level of endothelial progenitor cells (EPCs) and lower level of FMD than those of control group, ameliorating after CPAP treatment. Considering a circulating number of EPCs higher in children than adults, activation of factors involved in recruitment and addressing EPCs could lead to relevant differences in endothelial function impairment among children with OSAS.

Furthermore, Gozal et al. [37] observed that adenotonsillectomy did not lead to resolution of endothelial damage in children with a strong family history of cardiovascular diseases. If this hypothesis is confirmed by further studies, OSAS will become a major trigger for vascular dysfunction in children genetically predisposed to. These considerations lead to the improvement of an early detection of OSAS and endothelial dysfunction in children.

FMD is a favourable and noninvasive technique in order to evaluate endothelial function, although very young children could not be fully evaluated by FMD technique because they are often not cooperative. For this reason, there are few studies on basal values of FMD of brachial artery in healthy and ill children suffering from OSAS and obesity.

Our study, although with the limit of a small sample, aimed to evaluate such a correlation according to the real social and health impact of such a disease. For this reason, we considered the link among FMD, AHI, and BMI percentile.  Figure 1 put on evidence of a statistically significant correlation between FMD and AHI. This means that there is a progressively reduction of FMD values with the worsening of AHI ones, that is, a deterioration of endothelial function: arteries become more stiff and less able to act as normal. For this reason, we can affirm that OSAS could lead to a premature endothelial dysfunction even in the early childhood, and an attempt to reduce early respiratory impairment [38] should be always achieved in these patients, although being hard to manage [39]. At the moment, elective treatment for adults and children who are not candidates to adenotonsillectomy, is CPAP, even lifelong, with all its social, familial, and economic limitations [39]. The results seem to be in accordance with literature data [9].

Such a distinction has not been made in other studies and could become an important start for future researches perhaps using longitudinal ones along years, for a strict evaluation of pharmacological therapy.

## 6. Limitations

The small sample size is the first limitation of the present study, although the difficulty in enrolling such kind of little patients explains such a reduced number of patients.

Furthermore, it has not been possible to observe whether the reduction of desaturation episodes and snoring would correct FMD values in this population: a dedicated and more population-wide trial would be designed in order to develop such points and overcome limitations.

## 7. Conclusions

The results coming from our research, in line with the few literature data, are limited by the smallness of the sample. Nevertheless, our work prepares the beginnings for future researches through which, using longitudinal data, it will be possible to analyze the same subjects with FMD technique after a good pharmacological, surgical, orthodontic, or even dietary treatment.

## Figures and Tables

**Table 1:** General characteristics of study population.

	SDB Group 1 *N* = 23	Control Group 2 *N* = 32	*P*
Age (years)	9 ± 3	10 ± 3	NS
Gender (M/F)	12/11	14/18	NS
FMD (%)	8.4 ± 2.7	11.1 ± 3.0	0.001
BMI (kg/m^2^)	19 ± 3	18 ± 3	NS
BMI percentile (%)	58 ± 26	45 ± 22	0.048
OSAS degrees			
(i) Snoring	15 (65.2%)	—	
(ii) Minimum OSAS	4 (17.4%)	—	
(iii) Mild OSAS	4 (17.4%)	—	

SDB: sleep disordered breathing; FMD: flow-mediated vasodilation; BMI: body mass index; OSAS: obstructive sleep apnoea; NS: not significant.
